# Behaviour Hallmarks in Alzheimer’s Disease 5xFAD Mouse Model

**DOI:** 10.3390/ijms25126766

**Published:** 2024-06-20

**Authors:** Mafalda Soares Pádua, José L. Guil-Guerrero, Paula Alexandra Lopes

**Affiliations:** 1 CIISA—Centro de Investigação Interdisciplinar em Sanidade Animal, Faculdade de Medicina Veterinária, Universidade de Lisboa, 1300-477 Lisboa, Portugal; mafaldapadua@fmv.ulisboa.pt; 2 Laboratório Associado para Ciência Animal e Veterinária (AL4AnimalS), Faculdade de Medicina Veterinária, Universidade de Lisboa, 1300-477 Lisboa, Portugal; 3 Departamento de Tecnología de Alimentos, Universidad de Almería, 04120 Almería, Spain; jlguil@ual.es

**Keywords:** Alzheimer’s disease, transgenic mice, 5xFAD model, behaviour, cognitive decline, sensorial impairment

## Abstract

The 5xFAD transgenic mouse model widely used in Alzheimer’s disease (AD) research recapitulates many AD-related phenotypes with a relatively early onset and aggressive age-dependent progression. Besides developing amyloid peptide deposits alongside neuroinflammation by the age of 2 months, as well as exhibiting neuronal decline by the age of 4 months that intensifies by the age of 9 months, these mice manifest a broad spectrum of behavioural impairments. In this review, we present the extensive repertoire of behavioural dysfunctions in 5xFAD mice, organised into four categories: motor skills, sensory function, learning and memory abilities, and neuropsychiatric-like symptoms. The motor problems, associated with agility and reflex movements, as well as balance and coordination, and skeletal muscle function, typically arise by the time mice reach 9 months of age. The sensory function (such as taste, smell, hearing, and vision) starts to deteriorate when amyloid peptide buildups and neuroinflammation spread into related anatomical structures. The cognitive functions, encompassing learning and memory abilities, such as visual recognition, associative, spatial working, reference learning, and memory show signs of decline from 4 to 6 months of age. Concerning neuropsychiatric-like symptoms, comprising apathy, anxiety and depression, and the willingness for exploratory behaviour, it is believed that motivational changes emerge by approximately 6 months of age. Unfortunately, numerous studies from different laboratories are often contradictory on the conclusions drawn and the identification of onset age, making preclinical studies in rodent models not easily translatable to humans. This variability is likely due to a range of factors associated with animals themselves, housing and husbandry conditions, and experimental settings. In the forthcoming studies, greater clarity in experimental details when conducting behavioural testing in 5xFAD transgenic mice could minimise the inconsistencies and could ensure the reliability and the reproducibility of the results.

## 1. Introduction

Human ageing is the progressive accumulation of changes with time that are associated with or responsible for the ever-increasing susceptibility to many metabolic disorders, including among others, the neurodegenerative diseases, such as Alzheimer’s [1,2,3]. Over the last two decades, the percentage of the European population over 64 years old has increased by 5%, escalating from a total of 16% to 21%. In Portugal and Italy, the European countries with the oldest populations, this percentage rounds approximately 24% [4]. It is estimated by the World Health Organization (WHO) that, by 2050, the population aged 60 years old and more will reach a total of 2.1 billion people, which portrays an increment of more than 100% since 2020, when the reported number was 1 billion [5]. As a result, the proportion of people living with dementia and Alzheimer’s disease (AD) has also increased rapidly, with almost 10 million new cases emerging each year, and it is predicted to afflict a total of 139 million people until 2050, which represents an increase of more than 150% since 2019 [6,7,8]. Similarly, a fast growth in the prevalence of these disorders has been reported in Portugal [9,10], placing this health problem as a major global public health concern that needs to be urgently addressed [7,8].

AD is a multifactorial and multigenic neurodegenerative disorder that currently accounts for the majority of dementia cases worldwide [6,8,11,12,13]. Alzheimer’s patients consistently exhibit synaptic loss associated with massive neuronal loss, amyloid plaques, and neurofibrillary tangles in the brain, mostly in the cortical and the hippocampal regions, thus shaping the pathological hallmarks of this disease [6,11,12,13,14,15,16]. β-amyloid plaques evolve from an imbalanced cleavage of the amyloid precursor protein (APP) or a decreased degradation of β-amyloid protein, entailing an increased concentration of this peptide that leads to the formation and deposition of oligomers in the extracellular spaces. These are inductors of neurotoxicity in the brain, due to the many active chemical groups that remain exposed [6,11,17,18]. On the other hand, neurofibrillary tangles arise from the microtubule-associated tau protein that becomes hyperphosphorylated, leading them to dissociate from these structures, and consequently accumulate and aggregate in the somatodendritic compartment of neurons, compromising the stability of this cell’s structure and function [11,16,17]. On the whole, this neurodegenerative disease is outlined by gradual and progressive memory impairment, deterioration of other cognitive capacities, and inability to carry out daily routine activities [12,19].

Despite the substantial progress in AD knowledge since the first report by Alois Alzheimer in 1907 [14], there is still no effective treatment to fully reverse the underlying causes of this disorder [6,7,20]. Therefore, the scientific research concerning the pathological pathways, diagnostic assessment, and disease-modifying treatments remains a priority.

In a first approach, the best way to study a human disease and its underlying molecular mechanisms is by performing experimental clinical trials directly on humans; however, concerning AD, this approach is considered unethical and impractical because the human brain is highly unapproachable. Post-mortem analysis, if available, poses the biggest limitation regarding physiological systems that are no longer active, and thereby are not very fruitful [19,21,22]. Therefore, several animal models mimicking AD have been developed and used to provide further enlightenment on the pathophysiological features of the emergence and progression of this disease, as well as on the potential of novel diagnostic and therapeutic approaches [19,21,23,24,25,26]. Even though none of the existing animal models fully replicates human AD, in terms of its pathological, biochemical, and behavioural characteristics, the most accepted and widely used is the transgenic mouse model [21,22,24,27,28,29]. To date, more than two hundred mouse models have been developed based on AD-linked mutations and their different combinations [30]. This review focuses on one of the most commonly used and commercially available AD mouse model, the 5xFAD transgenic mouse, highlighting its main features in what concerns brain morphological characteristics and the resulting behavioural impairments, related to motor abilities, sensory inputs, cognitive decline, and psychological symptoms.

## 2. The 5xFAD Mouse Model

### 2.1. Rationale for the 5xFAD Mouse Model Development

The genesis of the 5xFAD mouse model is derived from the need to create a model that truly replicates the genetic and neuropathological features of familial AD (FAD), which constitutes a small but significant fraction of AD cases. FAD is primarily caused by mutations in the genes encoding APP and presenilins (PSEN1 and PSEN2), leading to increased production of β-amyloid peptides, particularly the highly aggregation-prone β42 isoform [31,32].

The 5xFAD model was designed to incorporate multiple FAD mutations to exacerbate β-amyloid aggregation and to accelerate disease onset, mirroring the aggressive pathology observed in human FAD cases. The 5xFAD transgenic mouse was developed in 2006 and simultaneously overexpresses the human APP gene encoding three FAD mutations, namely the Swedish (KM670/671NL), the London (V717I), and the Florida (I716V) mutations), and the human PSEN1 gene concealing two FAD mutations (the M146L and the L286V) [33]. These mutations are known to increase the production and aggregation of β-amyloid peptides, leading to the formation of amyloid plaques and subsequent neurodegeneration [33]. Both transgenes were inserted into the mouse genome under the control of neuronal promoters, the mouse Thy1 promoter, to drive expression specifically in neurons, mimicking the cellular localisation observed in human AD brain [34].

Upon generation, the 5xFAD mouse model underwent extensive validation and characterisation to confirm the fidelity of transgenes expression and the recapitulation of AD pathology. Histological analyses revealed the presence of β-amyloid plaques, synaptic dysfunction, neuroinflammation, and cognitive deficits, closely resembling the neuropathological hallmarks of human AD. Behavioural assessments further confirmed progressive cognitive decline, providing evidence of functional deficits associated with AD pathology [34].

This mouse model owes its popularity and wide use to the fact that it is commercially accessible, which reduces the cost and time to obtain this strain. In addition, AD manifestations arise rapidly and aggressively, reducing the costs and the time associated with the animals’ housing, which is beneficial on the development of therapeutic and preventive approaches [35,36].

In the 5xFAD mice, brain morphological evidence of AD starts to emerge at a very early age (1.5 months old) with intense amyloid pathology characterised by increased levels of β-amyloid and intraneuronal deposits. At 2 months of age, extracellular deposits start to form, primarily in the subiculum, deep cortical layers, and frontal cortex, progressing with age, and eventually spreading to much of the cortex, subiculum, and hippocampus [33,37,38,39,40] regions. Simultaneously to β-amyloid extracellular deposits at 2 months old, the neuroinflammation by reactive astrocytes and microglia starts to be detected, presenting an age-dependent progression as well as similar spread pattern [33,38,39,40]. Strongly associated with intra- and extracellular β-amyloid deposits, dystrophic neurites become visible at 3 months old, and are followed by deficient synaptic transmission at 4 months old, matching the onset of neuronal decline that becomes massive at 9 months of age [33,37,40,41].

Behavioural impairments, as a consequence of these structural alterations in the brain, also begin to appear in 5xFAD mice early stages and progress in an age-dependent manner, conditioning almost all of their day-to-day activities in more advanced stages of the animals’ life.

### 2.2. Cognitive and Physiological Impairments Conditioning Behaviour

Given the behavioural impairment found in AD patients, the evaluation of this parameter as well as the variables’ conditioning are also crucial in AD mice models. To date, a wide range of behaviour tests have been developed to assess behavioural parallelism between mice and humans across multiple settings. The repertoire of behavioural experiments with 5xFAD mice is extensive, and therefore, in this review, we categorise them into 4 groups: those that measure motor ability, those that test sensory performance, those that assess learning and memory skills, and finally, those that evaluate the psychological condition and the social behaviour of individuals.

#### 2.2.1. Motor Capacity

Just like in AD patients, 5xFAD mice display several motor impairments [42]. These events begin around 9 months of age, which coincides with the phase of drastic neuronal decline (affecting the primary motor cortex and complementary motor areas) due to the massive accumulation of β-amyloid plaques [33,41].

**Figure 1:**
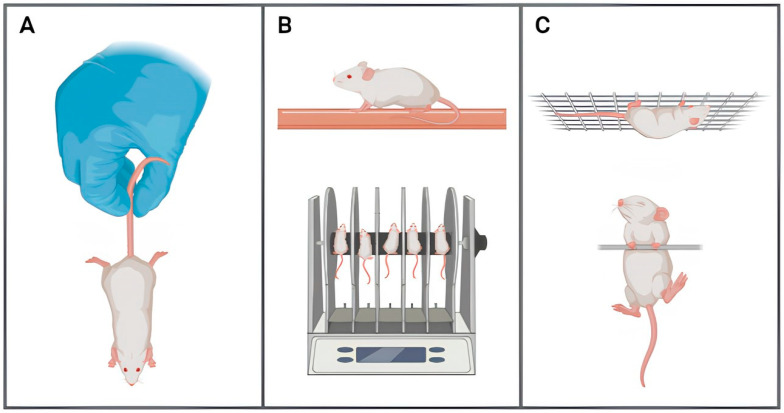
Illustration of the different tests applied to 5xFAD mice to assess their motor ability, regarding: (**A**) agility and reflex movements by tail suspension test; (**B**) balance and coordination by balance beam test and rotarod test, respectively; (**C**) skeletal muscle function by wire and grid suspension tests. Illustration generated using BioRender.com (accessed on 12 April 2024).

The agility and reflex movements of these mice are typically assessed by a tail suspension test, in which rigidity, stretching of paws, and hind- and forelimb clasping are monitored while the mouse is held by its tail (Figure 1A). There is almost consensus that mice up to 9 months of age are still agile and show normal reflexes [43,44,45]; however, Richard et al. [43] found changes in these parameters at 5 months of age. It is widely accepted that these animals display abnormal reflexes with more immobility, rigidity of the paws, and clasping of the hind- and forelimbs aged 9 months old [41,46,47,48,49,50,51]. The only exception is the one reported by Medina-Vera et al. [45], who found no variation in these variables in mice aged 12 months old.

The steady state of balance and coordination of these mice is assessed by rotarod test and balance beam test (Figure 1B) and is sometimes complemented by gait analysis, in which fall latency is recorded and sometimes combined with foot placement accuracy and step symmetry [52]. Regarding agility and reflex movements, 5xFAD mice do not suffer any balance or coordination disabilities until approximately 9 months of age [41,43,44,46,53,54,55,56,57]. From then on, those deficiencies appear to be age-related with progressive deterioration [41,46,49,56,57,58,59]. The few exceptions are those reported by Sawmiller et al. [60], who found loss of balance and coordination in 6-months-old mice, and also by Oblak et al. [61], who not only found no impairments of balance or coordination, but were able to observe improvements in those parameters at 6 and 12-months-old 5xFAD mice compared to their wild-type counterparts.

The skeletal muscle function of 5xFAD mice can be assessed by two distinct hanging procedures: wire and grid suspension tests, which evaluate forelimb and fore-plus hind-limb grip strength, respectively (Figure 1C) [62]. It gathers absolute consensus that, at 9 months of age, these mice do not exhibit deficits in skeletal muscle function and performance [41,43,48,49,54,55,56,58,63]. From then on, the majority of reports show impairments depicted by the shortening of hanging times [41,43,48,49,54,55,58,59,63]. O’Leary and colleagues [56], discordantly, suggest that these shortcoming performances occur later on, around 13–16 months old.

The locomotor function of rodents is typically assessed in almost all behaviour tests, regardless of the parameter which the test focuses on, in order to verify whether this functional aspect is limiting or conditioning the outputs.

The conflicting reports from different laboratories in the available literature covering motor skills are summarised in Table 1.

#### 2.2.2. Sensory Capacity

Similarly to AD patients, the 5xFAD mice display several sensory-associated impairments [64]. These impairments arise from the progression of β-amyloid peptide deposition and neuroinflammation through different regions of the brain, such as the hippocampus, olfactory bulb, visual cortex, and auditory system. All of these phenomena together lead to the degradation of certain elements of such anatomical structures and/or metabolic dysfunctions [65,66,67]. Unfortunately, the studies associating these physiological/sensory malfunctions to behavioural impairments are scarce and, therefore, it is mandatory to assess this affinity further.

The taste profile is altered in individuals suffering from AD [68,69]. This variable was evaluated in 5xFAD mice by Medina-Vera and colleagues [45] using a saccharin and sucrose preference test. It was found that these animals demonstrate, from 5 months old, a preference for a palatable sweet compound, given the fact that they exhibit a lower sucrose intake (below anhedonia level, as the inability to experience joy or pleasure), inducing a discrepancy in sucrose versus saccharine preference.

The auditory function of AD patients is impaired [70]. In mice, the behavioural performance associated with this dysfunction is widely assessed by acoustic startle reflex and prepulse inhibition tests [71,72]. Consistently, 5xFAD mice demonstrate hearing impairments; however, the life stage in which these impairments arise is not consensual, as O’Leary and colleagues [49,73] and Weible et al. [74] observed it as early as 3 months old, whereas Medina-Vera et al. [45] observed a tendency towards this deficiency only at 12 months of age.

The olfactory function is severely diminished in people with AD [75]. A wide variety of behavioural tests was designed to evaluate rodents’ olfactory function, including discrimination, preference, and avoidance tests as well as habituation and dis-habituation tests, and buried food pellet recovery tests [76]. Nonetheless, there is a great discrepancy regarding olfactory dysfunction in 5xFAD mice, given that some studies have found impairments in individuals aged around 4–6 months, and never before that [38,77,78,79], while other reports have assessed this sensory perception at the same age or in later stages of life, and never found any dysfunction [45,80,81,82,83].

The visual performances, in particular visual acuity and contrast sensitivity, are both diminished in AD patients [84]. In rodents, these parameters can be assessed by the optokinetic system and/or visual water task, and black and white transition system, respectively [85,86,87]. After examining the scarce studies carried out so far, there is unanimity regarding the existence of deficiencies in the visual acuity of 5xFAD mice [66,88], even though Criscuolo and colleagues [88] found a significant reduction in visual acuity from 1.5 months old, whereas Zhang et al. [66] reported no meaningful differences before 6 months of age. Regarding the contrast sensitivity, no discrepancies were registered at 6-months-old 5xFAD mice [66].

The conflicting reports from different laboratories in the available literature covering sensory-associated impairments are summarised in Table 2.

#### 2.2.3. Learning and Memory Capacity

The most studied behavioural impairments in AD mice models are associated with learning capacity and memory ability, as these are amongst the first and the most severe disabilities experienced by AD patients [89]. In 5xFAD mice, these impairments emerge at 4–6 months of age and are strongly associated with the spread of β-amyloid deposits and neuroinflammation through the medial temporal lobe and the subsystems linked to it, which include critical structures for learning capacity and memory function, such as the hippocampus, the prefrontal and the parietal cortex, as well as the amygdala [33,90,91].

**Figure 2:**
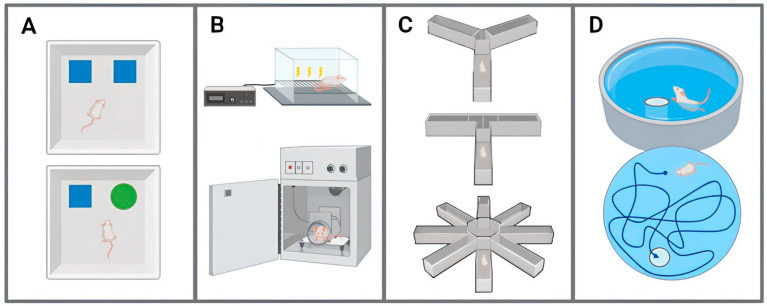
Illustration of the different tests applied to 5xFAD mice to assess their learning and memory ability, regarding: (**A**) visual recognition memory by novel object recognition (NOR) test; (**B**) associative learning and memory by contextual and/or cued fear-conditioning test; (**C**) spatial working memory, which includes T-maze, Y-maze, and radial maze tests; (**D**) spatial reference learning and memory by Morris Water Maze test. Illustration generated using BioRender.com (accessed on 24 April 2024).

The visual recognition memory of rodents is conventionally evaluated by the novel object recognition (NOR) test (Figure 2A). In this assessment, the time spent exploring a novel object (which is a behaviour that these animals have a natural propensity for) is compared to the time devoted to exploring an object which they are already familiar with [92]. It is almost consensual that 5xFAD mice demonstrate unimpaired visual recognition memory until 4 months of age, showing a clear preference for the exploration of the novel object compared to the familiar object, after which the display of those deficits begins, with no differences being observed regarding the time spent exploring either the novel object or the familiar object [51,53,93,94,95,96,97,98,99,100,101,102,103,104]. In contrast to these observations, Huttenrauch and colleagues [58] reported no deficiency in NOR test at 12 months old mice.

The associative learning and memory of mammals are frequently assessed by contextual and/or cued fear-conditioning test (Figure 2B). In this test, animals are initially exposed to a conditioned stimulus followed by an aversive unconditioned stimulus, and upon re-exposure to the conditioned stimulus. The fear memory is estimated through the freezing behaviour exhibited [105,106]. It is consensual that 5xFAD mice exhibit no signs of associative learning and memory deficits before 5 months old. From this age on, mice start to develop learning and memory impairments [48,60,107,108,109,110,111,112,113,114]. Divergently, Bhattacharya and co-workers [55] found no evidence of associative learning and memory deficiencies when testing 7-months-old animals, similarly to Forner and colleagues [40] who did not find any relevant impairment in mice up to 18 months old.

The spatial working memory of mice is often assessed by T- and Y-maze tests but, sometimes, it is also assessed by cross or radial maze tests (Figure 2C) [115,116]. In all of these tests, the spontaneous alternation of a chosen entry arm is assessed, considering that it is a natural tendency of rodents to prefer the exploration of a novel area over a familiar one [115,116]. Most studies evaluating this parameter in 5xFAD mice have concluded that the spatial working memory impairments typically emerge in individuals around 4–6 months old, but never before this age, and progressively decline with age [33,41,44,46,51,54,58,59,63,117,118,119,120,121,122,123,124,125,126,127]. However, a report exists about the onset of this dysfunction in earlier stages of life (in mice aged 2.5 months old) [128]. In addition, and contradicting the aforementioned literature, some reports found no deficiency in this domain around 4–6 months old or even in later stages of life, as the case of 12–14-months-old mice [45,61,97,103].

The spatial reference learning and memory are the behavioural metrics that have been most extensively researched in 5xFAD mice by the well-established Morris Water Maze test (Figure 2D) [129]. In this test, the learning skills of mice are assessed over several trials in which the latency to locate a hidden platform is recorded each time (normally decreasing in subsequent trials), after which the hidden platform is removed, and the memory skills are assessed through the amount of time spent in the quadrant where the platform was previously located [129]. The abundance of reports with those tests leads to a considerable variation in the outcomes. Although all the performed studies have found impairments in the spatial reference learning and memory of 5xFAD mice, with the exception of the one carried out by Medina-Vera and colleagues [45], who did not find any dysfunction, the onset age of these limitations is rather controversial. Some researchers reported dysfunctions in these parameters at very early stages of life, around 1–3 months of age [120,130,131,132], while the large majority of studies indicates that such impairments start to manifest at 4–6 months old, and never before [43,60,66,77,97,99,103,107,114,123,124,126,133,134,135], progressing in an age-dependent manner [48,49,58,59,121,122,136,137,138]. Nonetheless, various studies have identified these impairments emerging at a more advanced age, in approximately 9–12-months-old mice [47,50,112,139].

The conflicting reports from different laboratories in the available literature covering learning and memory are summarised in Table 3.

#### 2.2.4. Neuropsychiatric-like Behaviours

Given that AD patients exhibit psychological symptoms [140] as well as social cognition impairments [141], it is essential to evaluate these AD-related pathologies using AD transgenic mouse models [142]. These impairments derive from the progression of β-amyloid plaques and neuronal decline in the prefrontal cortex and hippocampus, leading to apathy-, anxiety-, and depressive-like behaviours as well as abnormal social interactions and exploratory activity in 5xFAD mice [125,143].

**Figure 3:**
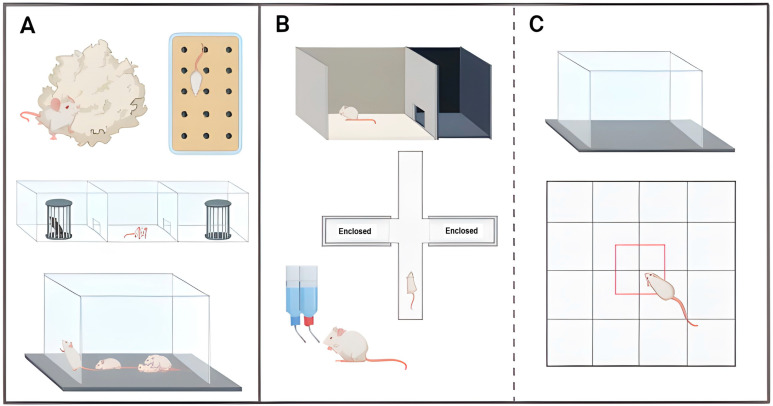
Illustration of the different tests applied to 5xFAD mice to assess their neuropsychiatric-like behaviours, regarding: (**A**) apathy-like behaviour, which includes nest-building and marble-burying tests as well as social interaction tests; (**B**,**C**) anxiety- and depressive-like behaviours, which include light/dark box, elevated plus maze, open field, and sucrose preference tests; (**C**) exploratory behaviour open field test. Illustration generated using BioRender.com (accessed on 26 April 2024).

Apathy-like behaviour is characterised by a diminished motivation to pursue goal-oriented actions or engage in socio-emotional interactions [125]. This neuropsychiatric-like behaviour is generally assessed by nest-building, burrowing, and marble-burying tests, in which the drive to engage in natural daily activities is evaluated, along with the social interaction test, in which the assessment is focused on the tendency to participate in social exchanges and the way they interact (Figure 3A) [125,144,145]. It is widely recognised that signs of apathy-like behaviour begin to manifest at 6-months-old 5xFAD mice and progressively deteriorate with age. 5xFAD mice start exhibiting reduced interactions with conspecific [50,51,82,101], coupled with atypical social behaviour during such interactions [82,137], alongside poor quality in nest construction [123,125,127,146], and poor performance in burrowing and marble burying [125]. Distinctly, only Liu and colleagues [123] found no deficiencies in the performance of burying marbles in 6-months-old 5xFAD mice.

Anxiety and depression are common and debilitating symptoms, which can profoundly impact an individual’s daily life [147]. Anxiety-like behaviour is commonly assessed through open field test, elevated plus maze test, and light/dark box test (Figure 3B,C) [147,148]. In turn, depressive-like behaviour is currently evaluated by sucrose preference test (Figure 3B) [148]. The findings regarding anxiety- and depressive-like behaviour in 5xFAD models are contentious. While certain reports indicate a reduction in anxiety-like behaviour, other studies have found no changes or even an increase in both anxiety- and depressive-like behaviours. It is broadly acknowledged that 5xFAD mice exhibit no changes in behaviour related to anxiety or depression until they reach approximately 5–6 months of age [40,41,43,46,47,56,60,123,130]. Regarding anxiety, and from that point onward, few authors consistently reported no alterations in behaviour even in later stages of life [56,137]. Whereas a great number of reports have noted a gradual reduction in anxiety-like behaviour [40,41,43,44,47,55,58,59,104,127,149,150], a small number of studies have observed an increase in such behaviour [49,50,51]. Regarding depression, Medina-Vera and colleagues [45] mentioned depressive-like behaviour in 5xFAD mice from 5 months old with an age-related progression as the sucrose intake of the individuals was below the anhedonia criterion.

Exploration is a typical behaviour for rodents. Animals are naturally drawn to new stimuli, leading them to engage in extensive exploratory behaviour upon encountering an unfamiliar environment in order to collect new information about it [151,152]. The exploratory behaviour encompasses a wide array of activities, such as scanning, leaning, sniffing, walking, rearing, jumping, and digging, and is predominantly evaluated by the open field test (Figure 3C) [151,153]. In this test, the frequency and the time consumed in each one of the activities as well as the distance moved, speed, and time spent in the activities are monitored [153]. The reports related to the exploratory behaviour of 5xFAD mice are characterised by significant inconsistencies and controversy. Both the level of activity exhibited by these mice and the age associated with the appearance of these behavioural changes do not have a consensus. Within the extensive existing literature, mice demonstrate normal levels of exploratory activity until 6–9 months of age [40,41,44,46,56,93,94,95,103,127], after which deviations from wildtype mice exploratory activity are usually detected. The few exceptions to this are some authors that describe earlier onsets at 4–5 months old [101,126] as well as later onsets at 12–18 months old [40,56], or even no onset at all [41]. Concerning the variation observed in the exploratory activity of 5xFAD mice, some studies indicate a reduced activity level (referred to as hypoactivity) [40,49,56,57,60,126,127], while other authors report an increased activity level (referred to as hyperactivity) [48,61,101,103,104,137]. Despite the divergence on the activity levels among mice, there is a consensus that animals devote less time to rearing compared to their non-transgenic littermates [49,56,57,60,61,127].

The conflicting reports from different laboratories in the available literature covering neuropsychiatric-like symptoms are summarised in Table 4.

## 3. Final Considerations and Forthcoming Directions

Behaviour testing in laboratory animals is vital for understanding how the brain supports sensory motor function, cognition, emotion, and many other processes [154]. Hence, it is crucial for neurodegenerative conditions, like AD, to understand how brain function is altered across different disease states.

Despite the popularity that behavioural phenotyping has gained over the past three decades, conflicting reports from different laboratories, as those described throughout this review, are a significant challenge in translating research findings into a deeper understanding on AD human conditions [155,156]. The substantial variability and inconsistency in the results have raised numerous discussions regarding the reproducibility, the validity, and the reliability of those studies [154,157,158,159]. Although there are numerous well-established tests for assessing various behavioural aspects, minor methodological differences can dramatically impact the outcomes [150].

Often, the inconsistencies are attributed to a combination of factors that can be linked to the animals, housing and husbandry conditions, and/or to the experiment. Animal factors are associated with species, strain, substrain, and background of the animals in use, as well as gender [36,156,157,158,160]. For females, this also includes consideration for oestrous cycle phase [156,157]. The behaviour of animals can also be influenced by housing factors, like the microbiological status of the facility [36]. The composition of the gut microbiota impacts on β-amyloid deposition, potentially delaying or enhancing behavioural phenotypes [161]. Moreover, the housing density and the presence of enrichment in animal cages are additional factors that can impact behaviour [156,158,160]. For instance, mice singly housed or without exposure to enrichment exhibit poorer performance in cognitive and social tasks, alongside altered anxiety- and depressive-like behaviours [162]. Another important influencing factor is the type of diet provided, regarding its nutritional content and energetic value, along with the established feeding routine, whether it is ad libitum or another feeding scheme [156,157]. When it comes to experimental factors, there are a multitude of elements that can impact the behavioural performance of animals. The experimenter with expertise and training in handling and behaviour testing [157], gender [163], and familiarity with animals [164], will significantly impact the experimental outcomes. The season of housing and testing, together with the time of day during which the tests are conducted, are two additional factors that can markedly influence the experimental findings [157,160]. These may be due to the fluctuations in temperature and humidity perceived across different seasons or the animals’ endogenous circannual rhythms [165,166], as well as to the light/dark cycle along with the timing associated with the circadian rhythm of mice [167]. Additional factors, such as whether or not fasting protocols are implemented before the experiment [168], combined with the lighting conditions during the assay [169], and the sequence in which tests are performed [157,160], can also contribute to the potential variability in the findings. Another contributing factor to such variability is the scoring methodology employed in the experiment. The criteria for scoring, such as the number of entries or their duration, combined with whether these parameters are recorded manually or automatically, which can account for experimenter bias, are also relevant to consider [154,160].

Overall, it is important to not overlook the fact that different areas of behaviour are interconnected; therefore, any disruption in a particular behaviour can have repercussions on additional ones [139,156,170]. For instance, deficits in sensory–motor ability can confound findings related to cognitive functions (e.g., associative learning and memory assessed by an auditory-cued fear conditioning test involving mice with hearing impairments) and emotional behaviours (e.g., anxiety-like behaviour assessed through an elevated maze which is affected by deficits in visual ability). Additionally, hyperactivity may skew the outcomes of social behaviour assessment, as well as anxiety which can inhibit mice from performing in a range of other behavioural tests, as expected.

Hence, it is crucial to increase awareness of the multitude of factors that can impact mice behaviour. Researchers must, therefore, tailor their own experimental designs to account for these influencing factors. Specific strict protocols should exist and must be implemented for animals’ housing and husbandry when behavioural testing is intended. Moreover, greater transparency in reporting experimental settings and methodological details related to testing conditions, is necessary. Ideally, more detailed standard established protocols for the different behaviour tests should be developed to guarantee uniformity. Such clarity will allow fellow researchers to interpret and replicate results in the future, ultimately minimising the inconsistencies and enhancing the robustness and the reliability of preclinical studies.

## Figures and Tables

**Table 1:** Summary of the contradicting literature on the onset of different motor impairments in 5xFAD mice.

	Early Onset (1–3 Months)	Mid-Onset (4–8 Months)	Late-Onset (9–15 Months)	Absence
Abnormal agility and reflex movements	-	[43]	[41,46,47,48,49,50,51]	[45]
Loss of balance and coordination	-	[60]	[41,46,49,56,57,58,59]	[61]
Deficits in skeletal muscle function	-	-	[41,43,48,49,54,55,56,58,59,63]	-

**Table 2:** Summary of the contradicting literature on the onset of different sensory-associated impairments in 5xFAD mice.

	Early Onset (1–3 Months)	Mid-Onset (4–8 Months)	Late-Onset (9–15 Months)	Absence
Altered taste profile	-	[45]	-	-
Auditory dysfunction	[49,73,74]	-	-	[45]
Olfactory dysfunction	-	[38,77,78,79]	-	[45,82,83]
Impaired visual acuity	[88]	[66]	-	-

**Table 3:** Summary of the contradicting literature on the onset of different learning and memory impairments in 5xFAD mice.

	Early Onset (1–3 Months)	Mid-Onset (4–8 Months)	Late-Onset (9–18 Months)	Absence
Impaired visual recognition memory	-	[53,93,94,95,96,97,99,101,102,103,104]	[51,98,100]	[58]
Impaired associative learning and memory	-	[60,107,108,109,110,111,113,114]	[48,112]	[40]
Impaired spatial working memory	[128]	[33,41,44,46,54,63,118,119,120,122,123,124,126]	[51,58,59,117,121,125]	[45,61,103]
Impaired spatial reference learning and memory	[120,130,131,132]	[60,66,77,97,99,103,107,114,122,123,124,126,133,134,135,136]	[47,48,49,50,58,59,112,121,137,138,139]	[45]

**Table 4:** Summary of the contradicting literature on the onset of different abnormal neuropsychiatric-like behaviours in 5xFAD mice (↑—increased, ↓—decreased).

	Early Onset (1–3 Months)	Mid-Onset (4–8 Months)	Late-Onset (9–18 Months)	Absence
Apathy-like behaviour	-	[50,101,123,127,146]	[51,82,125,137]	-
Anxiety-like behaviour	-	-	↑ [49,50,51]	[56,137]
		↓ [40,41,43,44,55,104,149,150]	↓ [47,58,59,127]	
Depressive-like behaviour	-	[45]	-	-
Abnormal exploratory behaviour	-	↑ [48,61,101,104]	↑ [103,137]	-
	-	↓ [60,126]	↓ [40,49,56,57,127]	
